# Prevalence of frailty in China: Protocol for systematic review and meta-analysis

**DOI:** 10.1097/MD.0000000000020079

**Published:** 2020-05-15

**Authors:** Qi Wang, Honghao Lai, Yunhua Wang, Jing Qi, Bei Pan, Jiancheng Wang

**Affiliations:** a School of Public Health of Lanzhou University; b Gansu Provincial Hospital, Lanzhou, China.

**Keywords:** community-dwelling elders, frailty, meta-analysis, prevalence, protocol

## Abstract

**Introduction:**

China is facing more and more enormous challenges including aging, the increase of social security costs and health care cost, decrease of labors, and how to keep elders’ capacity to function well and live independently. Healthy aging has become an extremely challenging issue. Frailty, a geriatric syndrome resulted from the declines of multiple physiological systems, characterized by malnutrition, exercise intolerance, dependence, longer bed rest, lower gaits peed, weakness, weight loss, anorexia, hip fracture, risk of falling, delirium, dementia, and keep indoors, has become one of the biggest challenges in facilitating healthy aging. Because the research of frailty just had started in recent years in China, the evidence regarding the prevalence of frailty among the Chinese population is scarce and just limited in certain area.

**Method:**

We will systematically search 7 electronic database including PubMed, Embase, Cochrane Central Register of Controlled Trials (CENTRAL), Web of science, MEDLINE, CNKI (Chinese National Knowledge Infrastructure), and CBM (Chinese Biological Medical Database) to identify studies that provide or potential available of data regarding to prevalence of frailty. Risk of bias of individual study will be assessed using 8-iterm critical appraisal criteria for prevalence or incidence studies. Data will be analyzed using STATA V.12.0 software.

**Conclusions:**

Our systematic review and meta-analysis will firstly pool the results from available studies to provide a comprehensive evidence for the prevalence of frailty. The results of this study will be submitted to a peer-reviewed journal for publication.

## 1 Introduction

Population is growing older rapidly in recent centuries. It is estimated that people aged older than 65 years will accelerate from 461 million to 2 billion by 2050.^[1,2]^ According to “2017 Statistical Bulletin of National Economy and Social Development”, people aged older than 60 years accounted for 17.3% (240.90 million) of the total population, and people older than 65 years accounted for 11.4% (158.31 million) of the total population in 2017 in China.^[3]^ As the first large country of population in the world, China is facing more enormous challenges along with the aging, including the increase of social security costs and health care cost, decrease of labors, and how to keep elders’ capacity to function well and live independently. Healthy aging has become an extremely challenging issue.

Frailty, a geriatric syndrome, resulted from the declines of multiple physiological systems, characterized by malnutrition, exercise intolerance, dependence, longer bed rest, lower gaits peed, weakness, weight loss, anorexia, hip fracture, risk of falling, delirium, dementia, and keep indoors, has become one of the biggest challenges in facilitating healthy aging.^[4,5,6]^ Fried frailty phenotype^[7]^ and Rockwood's Frailty Index^[8]^ are 2 widely used definition tools of frailty. Five physical phenotypic variables (unintentional weight loss, low activity, exhaustion, slow gait speed, and weakness) were used to recognize frailty through Fried frailty phenotype, and older people were classified as frail (≥3 variables), pre-frail (1–2 variables), robust (0 variables). Frailty Index including 92 variables of symptoms, chronic disease (including depression and cardiovascular diseases) and disability, signs, and abnormal laboratory values were used to define frailty.^[9]^ Rockwood et al^[10]^ considered frailty as the cumulative effect of deficits, so the more deficit individuals have the higher risk to onset frail they are. Understanding the prevalence of frailty can better help researchers and medical workers to manage frailty, and therefore to reduce rate of institutionalization and risk of mortality.

The prevalence of frailty among general population older than 65 years has been reported from 4% in a United States study to 27.3% in a Spanish study. The prevalence is higher in patients with cancer (42%) and nursing home (52.3%).^[11,12,13,14,15]^ Because the research of frailty just had started in recent years in China, the evidence regarding the prevalence of frailty among the Chinese population is scarce and just limit in certain area.

Our systematic review and meta-analysis study aims to systematically search available information on the prevalence of frailty among Chinese community-dwelling elders, and pool the results from available studies to provide a comprehensive evidence for the prevalence of frailty.

## 2 Method

Our study protocol is developed according to Preferred Reporting Items for Systematic review and Meta-Analysis Protocols (PRISMA-P) statement.^[16]^ And this protocol has been registered in the international prospective register of systematic review (PROSPERO). The registration number is CRD42018091964.

### 2.1 Search strategy

We will systematically search electronic database including PubMed, Embase, Cochrane Central Register of Controlled Trials (CENTRAL), Web of science, MEDLINE, CNKI (Chinese National Knowledge Infrastructure), and CBM (Chinese Biological Medical Database) without search date and publication language restriction. The following search terms will be used for PubMed:

#1: “Frailty”[Mesh]) OR “Frail Elderly”[Mesh]) OR frailty [Title/Abstract]) OR frail [Title/Abstract]))
#2: “prevalence”[Mesh] OR “incidence”[Mesh] OR “epidemiology ”[Mesh]
#3: “Chine ”[Mesh] OR Chinese [Title/Abstract]
#4: #1and #2 and #3

### 2.2 Inclusion and exclusion criteria

We will include studies as following criteria: community-dwelling old people (aged ≥65 years) living in China; any cross-sectional studies or prospective studies that provide or potential available of data regarding to prevalence of frailty; defined frailty using Fried phenotype, Rockwood's Frailty Index, or any other modified versions among Chinese community-dwelling elders.

We will exclude studies included old people with selected diseases, such as cancer, cognitive impairment, depression, and Parkinson disease. We will also exclude randomized control trails, review articles, and conference abstracts.

### 2.3 Study selection

We will perform screen process using an online screening tool “rayyan.” A pilot test will be conducted initially to ensure high inter-rater reliability among the reviewers. And then 2 independent reviewers will screen all the searched bibliographic records through title and abstract according to eligibility criteria. Any potentially capable records will move on to full-texts screen. To illustrate the study selection process, we will also adhere to PRISMA guidelines to form flow diagram.^[17]^

### 2.4 Data extraction

After finish of the study selection, we will conduct a standard data abstraction sheet using Microsoft Excel 2010 to collect data of interest. One reviewer will extract the following characteristics: the first author, year of publication, name of cohort if any, country in which cohort were conducted, sample size, mean age, median weight, proportion of female, body mass index (BMI), race, comorbidities, social function score, smoke status (yes or no), drinking status (yes or no), living status (living alone or living with others), work status (current work, retired, or never work), frailty criteria, the number and percentage of participants according to frailty categories (frailty, prefrailty, and nonfrailty).

### 2.5 Risk of bias assessment

Methodological quality of all included cohort studies will be assessed according to 8-iterm critical appraisal criteria for prevalence or incidence studies^[18]^ including 3 subscales as follows: are the study methods valid? (Are the study design and sampling method appropriate for the research question? Is the sampling frame appropriate? Is the sample size adequate? Are objective, suitable, and standard criteria for measurement of the health outcome? Is the health outcome measured in an unbiased fashion? Is the response rate adequate? Are the refusers described?); What is the interpretation of the results? (are the estimates of prevalence or incidence with confidence interval and detail by subgroup, if appropriate), and What is the applicability of the results (are the study subjects and the setting described in detail and similar to those of interest to you). The methodological quality assessment will be conducted by 2 reviewers, and conflict will be resolved by a third reviewer. We will consider higher scores as higher quality, and the 8 as the maximum score possible.^[18]^

### 2.6 Meta-analysis

We will use STATA V.12.0 software (Stata Corporation, College Station, TX) to perform a meta-analysis. We will pool prevalence of frailty, prefrailty, and nonfrailty using random-effects model. Heterogeneity of treatment effects across trials will be assessed using the Q test with the DerSimonian-Laird method, and quantify heterogeneity using *I*^2^ statistics. We will consider 25%, 50%, and 75% as low, moderate, and high 25%, 50%, and 75% heterogeneity, respectively.^[19]^

### 2.7 Subgroup analysis

To examine the effect of study-level variables on the prevalence of frailty, we will perform priori subgroup analyses.^[20]^ We will examine studies identified as having a low risk of bias. And we also consider age, sex, district, and frailty assessment scale as subgroup factors if there are >2 studies for each subgroup factor.

### 2.8 Publication bias

We will draw a funnel plot to identify the possibility of publication bias using STATA V.12.0 software (Stata Corporation, College Station, TX).

Ethic

## Author contributions

**Conceptualization:** Jiancheng Wang.

**Data curation:** Bei Pan.

**Investigation:** Qi Wang.

**Methodology:** Bei Pan, Jiancheng Wang.

**Project administration:** Jiancheng Wang.

**Resources:** Qi Wang, Bei Pan.

**Supervision:** Qi Wang, Honghao Lai, Yunhua Wang, Jing Qi, Bei Pan, Jiancheng Wang.

**Validation:** Qi Wang, Honghao Lai, Yunhua Wang, Jing Qi, Bei Pan.

**Writing – original draft:** Qi Wang, Honghao Lai.
